# High infestation of invasive *Aedes* mosquitoes in used tires along the local transport network of Panama

**DOI:** 10.1186/s13071-019-3522-8

**Published:** 2019-05-27

**Authors:** Kelly L. Bennett, Carmelo Gómez Martínez, Alejandro Almanza, Jose R. Rovira, W. Owen McMillan, Vanessa Enriquez, Elia Barraza, Marcela Diaz, Javier E. Sanchez-Galan, Ari Whiteman, Rolando A. Gittens, Jose R. Loaiza

**Affiliations:** 1 Smithsonian Tropical Research Institute, Balboa Ancón, Republic of Panama; 2 The University of Texas, El Paso, TX USA; 3 Universidad Tecnológica de Panamá, Panamá, Republic of Panama; 4 University of North Carolina, Charlotte, NC USA; 5 Instituto de Investigaciones Científicas y Servicios de Alta Tecnología (INDICASAT AIP), Panamá, Republic of Panama; 6 Universidad de Panamá, Panamá, Republic of Panama

**Keywords:** *Aedes* mosquitoes, Arboviral vectors, Human-assisted transport, Vector surveillance, Disease control, Panama

## Abstract

**Background:**

The long-distance dispersal of the invasive disease vectors *Aedes aegypti* and *Aedes albopictus* has introduced arthropod-borne viruses into new geographical regions, causing a significant medical and economic burden. The used-tire industry is an effective means of *Aedes* dispersal, yet studies to determine *Aedes* occurrence and the factors influencing their distribution along local transport networks are lacking. To assess infestation along the primary transport network of Panama we documented all existing garages that trade used tires on the highway and surveyed a subset for *Ae. aegypti* and *Ae. albopictus*. We also assess the ability of a mass spectrometry approach to classify mosquito eggs by comparing our findings to those based on traditional larval surveillance.

**Results:**

Both *Aedes* species had a high infestation rate in garages trading used tires along the highways, providing a conduit for rapid dispersal across Panama. However, generalized linear models revealed that the presence of *Ae. aegypti* is associated with an increase in road density by a log-odds of 0.44 (0.73 ± 0.16; *P* = 0.002), while the presence of *Ae. albopictus* is associated with a decrease in road density by a log-odds of 0.36 (0.09 ± 0.63; *P* = 0.008). Identification of mosquito eggs by mass spectrometry depicted similar occurrence patterns for both *Aedes* species as that obtained with traditional rearing methods.

**Conclusions:**

Garages trading used tires along highways should be targeted for the surveillance and control of *Aedes*-mosquitoes and the diseases they transmit. The identification of mosquito eggs using mass spectrometry allows for the rapid evaluation of *Aedes* presence, affording time and cost advantages over traditional vector surveillance; this is of importance for disease risk assessment.

**Electronic supplementary material:**

The online version of this article (10.1186/s13071-019-3522-8) contains supplementary material, which is available to authorized users.

## Background

*Aedes aegypti* and *Aedes albopictus* mosquitoes are aggressive invaders of anthropogenic environments capable of spreading dengue virus (DENV) with high efficiency, and can trigger epidemics even at low population density [1]. Both mosquitoes are suspected vectors of emergent chikungunya (CHIKV) and Zika (ZIKV) viruses within the Americas, yet their role in the transmission cycle of local/regional epidemics remains poorly understood [2, 3].

Despite having different biogeographical origins, with *Ae. aegypti* originating from Africa [4, 5] and *Ae. albopictus* from Asia [6], these mosquitoes have converged on a similar ecology, ovipositing in man-made water containers and feeding on human blood. The invasive spread of both species means they now coexist across much of their widespread geographical range, where they compete for space and resources [7, 8]. For example, *Ae. aegypti* has been widely distributed across Panama since its introduction in the 17th to 18th century, while *Ae. albopictus* has expanded towards the border with Costa Rica since it was first reported in Panama City in 2002 [9]. Both *Aedes* species exhibit adaptive traits to exploit human environments, including the capacity of eggs to withstand desiccation over prolonged dry conditions [10]. The ability to withstand desiccation allows eggs oviposited in transported items such as used tires and decorative epiphyte plants to survive, invade and establish in new geographical areas. It is well established that *Ae. aegypti* and *Ae. albopictus* are passively transported in aircraft, boats and terrestrial vehicles including *via* the used-tire shipping industry [11, 12], which has facilitated repeated intercontinental migration events for both species [5, 11, 13].

With a few exceptions [14–16], there is little direct evidence for the infestation of *Ae. aegypti* and *Ae. albopictus* along local transport networks, although transportation is widely supported by mark-recapture [17] and studies of regional genetic structure [11, 18, 19] showing evidence for long-distance human-assisted migration outside their limited flight range. Species distribution models suggest that the rapid spread of *Ae. albopictus* across Panama since 2002 is best explained by the road network, rather than the climate, human population density or landscape use [9], with used tires as a potential means of dispersal. However, there is no supporting empirical data about the spatial pattern of *Aedes* occurrence along the primary transport network of Panama, including whether their presence can be linked to the density of roads. The high frequency of occurrence of *Ae. aegypti* and *Ae. albopictus* along Panama’s transport network would suggest that they are equally likely to disperse assisted by humans; conversely, the low frequency of spatial co-occurrence would imply that additional factors might shape their local spatial distribution. This information is required to predict potential changes in mosquito distributions that influence the transmission of viral pathogens and is important for disease prevention and control.

Here, we evaluate the potential role of the used-tire industry in *Aedes* dispersal across Panama by determining the infestation rate, and whether this can be linked to the local primary transport network. In addition, we determine whether either species is more prevalent along the highway, and if they are more likely to coexist spatially than in isolation. Finally, in addition to using traditional surveillance methods which are labor intensive and specialised, we evaluate the use of matrix-assisted laser desorption/ionization time-of-flight mass spectrometry (MALDI-TOF-MS) to identify species based on protein spectra [20, 21]. This method could be a potentially rapid and reliable tool for *Aedes* surveillance across Panama.

## Methods

### Mosquito sampling and data analysis

**Fig. 1:**
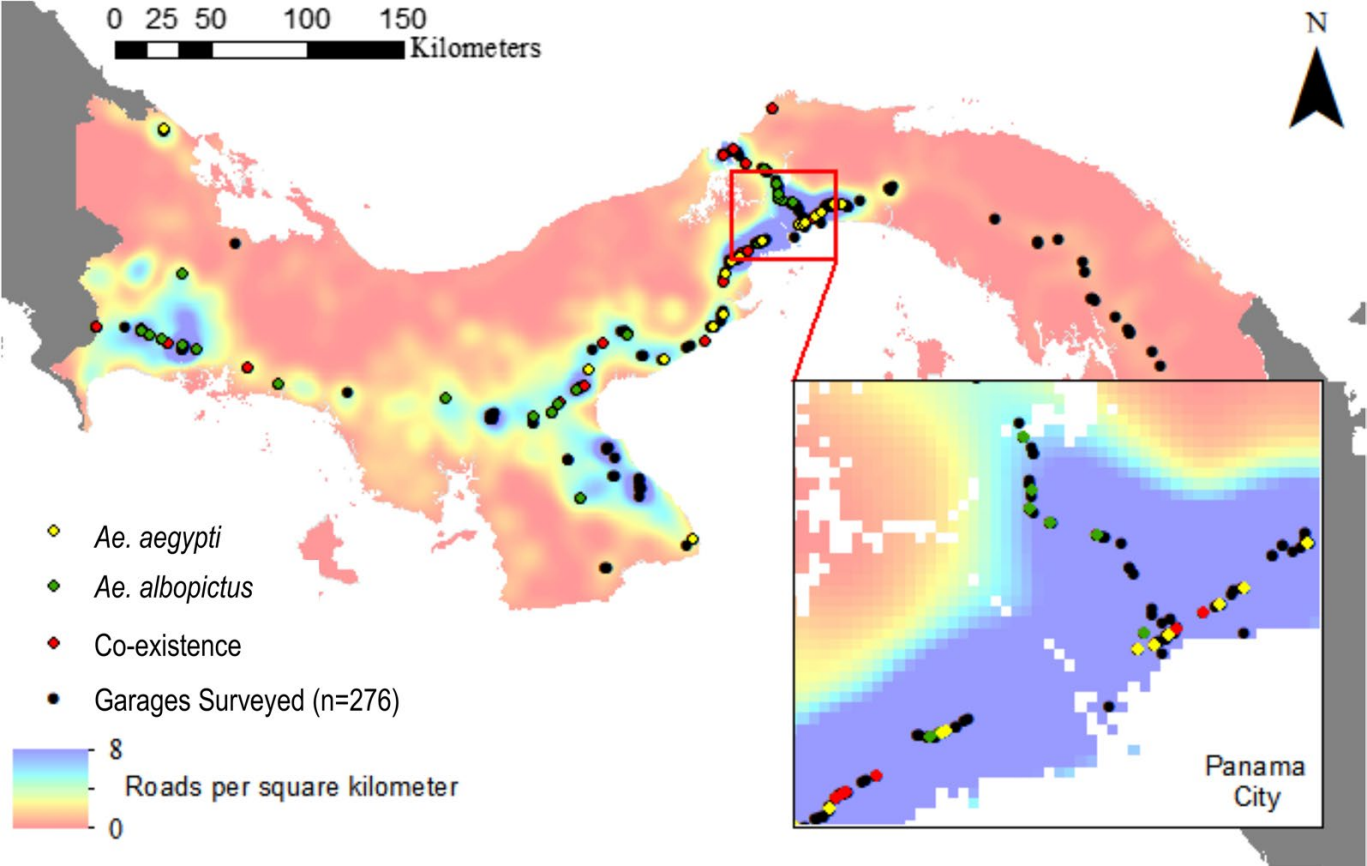
Map of Panama depicting the geographical locations of 276 garages trading used tires along the primary road network system of the country (black dots), including 79 sampled garages which were positive for the presence of *Aedes* mosquitoes. Yellow color dots represent the presence of *Ae. aegypti*; green color dots represent the presence of *Ae. albopictus*; red color dots represent the occurrence of both species (i.e. co-existence). The density of roads per square kilometer is depicted as a shading gradient of light and dark orange, yellow and blue colors. The map was created using ArcMap v.10.6 [28] with original data obtained from the GIS Laboratory, Smithsonian Tropical Research Institute 2011 (https://stridata-si.opendata.arcgis.com)

We first documented all existing garages that import and trade used tires along the primary transport networks of Panama by actively searching along the highways (Fig. 1, Additional file 1: Table S1). Geographical coordinates were recorded using a hand-held global positioning system (Garmin International, Olathe, KS, USA) and mapped as WGS84 data in ArcMap GIS (Environmental Systems Research Institute, Redlands, CA, USA). We then randomly chose and evaluated 84 (~30%) of all 275 recorded garages for the presence of *Aedes* mosquitoes with sampling occurring twice between 2016 and 2017. We sampled for mosquitoes by (i) actively searching for developing larvae in water-filled used tires in addition to the manual respiration of resting adults; and (ii) passively collecting eggs with oviposition traps placed in triplicate at the same garages. Since oviposition traps attract gravid adults if present in the area, they can be considered a more comprehensive sampling method that reduces false negatives and bias by inadequate sampling effort while searching for larvae. Collected mosquitoes were morphologically identified as larvae [22] or reared and identified as adults [23] before storage in absolute ethanol.

The rate of *Aedes* infestation in garages was calculated as the percentage of positive garages for the presence of *Aedes* mosquitoes out of the total number of garages surveyed in a particular year. We tested for an explicit pattern of spatial occurrence between *Ae. aegypti* and *Ae. albopictus* using a probabilistic model in the R package, co-occur [24, 25]. This model calculates the probability (*P*) that two species co-occur at a lower (*Plt*) or a higher (*Pgt*) frequency than the observed co-occurrence frequency if they were randomly distributed [26].

A kernel density estimation was performed on a shapefile of all roadways of Panama (GIS Laboratory, Smithsonian Tropical Research Institute, 2011) to determine the density of roads across the country since we expected the number of garages to increase with the number of roads. A random subset of 300 road density values were extracted from across Panama for comparison to the number of sampled garages within a 0.5 km radius from each extracted point. A linear regression was performed in STATA [27] to determine whether road density was a proxy for the number of garages found along the highway. Road density values were extracted at a 0.5 km resolution from the geo-coordinates of each garage surveyed for the presence of *Aedes* using ArcMap 10.6 [28]. A generalized linear model (GLM) analysis with a Poisson distribution was applied to this data to test whether the presence of only *Ae. aegypti*, only *Ae. albopictus* or the co-occurrence of both species was linked to the density of roads, with the latter modelled as the dependent variable.

### Matrix-assisted laser desorption/ionization mass spectrometry

With the goal of establishing a rapid identification approach for mosquito eggs that could save considerable time and effort in the process of evaluating mosquito prevalence for public health purposes, we assessed the utility of a MALDI-TOF-MS technique to classify eggs of *Ae. aegypti* and *Ae. albopictus*. These were gathered from garages by testing egg samples from roughly 20% (17 of 79) of all sampled garages showing single occurrence or co-infestation (Table 1). From randomly selected garages, half of the collected eggs were reared for adult mosquito identification, a process that could take 30 days per batch, while the other half were evaluated fresh with MALDI-TOF-MS. Roughly 30% of good quality and physically intact eggs from the three oviposition traps placed at each independent garage were chosen for analysis. Selected eggs were not adjacent to reduce the probability of sampling from the same egg-laying female. Eggs were dried and stored at −20 °C until mass spectrometry analysis.

**Table 1:** List of garages that trade used tires along the highways of Panama selected to identify eggs of *Aedes* mosquitoes with the MALDI-TOF-MS technique. The resulting outcome of species identification is compared to traditional methods of mosquito surveillance including active surveillance (AS) and oviposition traps (OVT). Additional details about each sampling site are provided in Fig. 1, Additional file 1: Table S1 and Additional file 3: Figure S1

No.	Province	Location	Lat.	Long.	Road density	MALDI-TOF-MS				Traditional approach				
						No. of eggs	*Ae. aeg*	*Ae. alb*	Result	*Ae. aeg* AS	*Ae. aeg* OVT	*Ae. alb* AS	*Ae. alb* OVT	Result
12	Chiriquí	David	8.527	− 82.836	0.698	13	9	4	Co-existence	5	32	8	21	Co-existence
18	Veraguas	La Mesa	8.202	− 81.186	0.963	29	0	29	*Ae. albopictus*	0	0	71	17	*Ae. albopictus*
20	Veraguas	La Mesa	8.117	− 80.967	1.912	14	5	9	Co-existence	0	0	1	0	*Ae. albopictus*
24	Coclé	Aguadulce	8.246	− 80.564	2.034	23	2	21	Co-existence	11	16	9	12	Co-existence
25	Coclé	Aguadulce	8.248	− 80.555	2.036	17	5	12	Co-existence	28	5	1	17	Co-existence
27	Coclé	El Roble	8.173	− 80.662	1.233	11	1	10	Co-existence	0	0	19	15	*Ae. albopictus*
29	Coclé	Penonomé	8.454	− 80.450	1.001	35	22	13	Co-existence	5	11	8	22	Co-existence
33	Coclé	Aguadulce	8.254	− 80.541	1.975	24	0	24	*Ae. albopictus*	0	0	28	14	*Ae. albopictus*
36	Panamá Oeste	San Carlos	8.467	− 79.966	1.283	9	0	9	*Ae. albopictus*	0	0	22	13	*Ae. albopictus*
39	Panamá Oeste	Capira	8.869	− 79.802	4.478	42	14	28	Co-existence	17	12	11	3	Co-existence
40	Panamá Oeste	La Chorrera	8.873	− 79.796	4.537	15	4	11	Co-existence	12	1	17	1	Co-existence
42	Panamá Oeste	La Chorrera	8.874	− 79.792	4.537	12	8	4	Co-existence	8	17	2	19	Co-existence
43	Panamá Oeste	La Chorrera	8.890	− 79.764	4.200	23	9	14	Co-existence	4	13	15	3	Co-existence
51	Panamá	Panamá	9.040	− 79.460	6.842	16	13	3	Co-existence	29	18	9	21	Co-existence
60	Panamá	Panamá	9.025	− 79.485	7.157	16	5	11	Co-existence	18	17	10	5	Co-existence
78	Colón	Cativá	9.360	− 79.830	2.247	11	8	3	Co-existence	30	4	7	2	Co-existence
83	Colón	Colón	9.339	− 79.880	1.349	2	0	2	*Ae. albopictus*	0	0	23	13	*Ae. albopictus*

*Abbreviations*: *Ae. aeg*, *Aedes aegypti*; *Ae. alb*, *Aedes albopictus*

Individual eggs were transferred to a 1.5-ml tube with 300 µl of deionized water and 300 µl of anhydride alcohol. Samples were vortexed and centrifuged for 2 min at 13,000× *rpm* to remove excess liquid. Dry eggs were mixed with 5 µl of formic acid, then 5 µl of acetonitrile and mechanically crushed with a metallic rod. Samples were submerged in an ultrasonic bath for one hour then centrifuged for 2 min at 13,000× *rpm*. One microliter of each sample was loaded in triplet on a MTP384 polished steel plate (Bruker Daltonics, Bremen, Germany) mixed with 1 µl of α-cyano-4-hydroxycinnamic acid matrix in 50% acetonitrile and 2.5% trifluoroacetic acid.

Mass spectrometry measurements were made with a UltrafleXtreme MALDI-TOF/TOF (Bruker Daltonics, Bremen, Germany) equipped with a 2 Khz SmartBeam™-II Nd:YAG laser (λ = 355 nm) used in positive polarization mode. All egg spectra were acquired with an automated method in the 2000–20,000 Da range in linear mode for peptides and intact protein detection. In some cases, the egg spectra were collected manually. Every spectrum represented the accumulation of 5100 shots with 300 shots taken at a time and with early termination if at least two of the peaks reached an intensity value of 10,000. The laser was set in random-walk mode with a raster spot of 50 μm, and laser power global attenuator offset of 32%. Spectra were collected with the FlexControl software (Bruker Daltonics, Bremen, Germany), calibrated using a pure sample of *Escherichia coli* protein extract as the bacterial test standard.

The software FlexAnalysis™ (Bruker Daltonics, Bremen, Germany) was used to evaluate the number of peaks and peak intensity after pre-processing mosquito egg spectra with smoothing and baseline subtraction. Flat-line spectra were immediately discarded and only spectra with at least one peak above an arbitrary intensity of 3000 considered for further analysis. Selected spectra were loaded into the ClintProTools™ program (Bruker Daltonics, Bremen, Germany) to perform statistical analysis and mathematical algorithms. Spectra were pre-treated with a standard workflow that included convex hull baseline subtraction, normalization to each of the spectrum’s own total ion count (TIC), recalibration using peaks that occur in at least 30% of spectra with a maximal peak shift of 1000 ppm, total average spectra calculation and average peak list calculation. The algorithm to classify mosquito eggs according to their respective spectra was a supervised neural network (SNN) [29]. The egg spectra from known mosquito colonies were used to train the SNN and create a classification model for identifying *Ae. aegypti* and *Ae. albopictus* spectra. To strengthen the model, the training dataset for *Ae. aegypti* also contained the spectra of eggs collected from a garage in Bocas del Toro, where national historical data for the past 20 years has shown only the presence of *Ae. aegypti*. The classification model was trained with 80% of the respective reference sets to determine the recognition capability with these known samples and the remaining randomly-selected 20% of spectra from each group was used to establish the algorithm’s cross-validation performance using 20 iterations (Additional file 2: Table S2). Subsequently, we proceeded to catalogue the taxonomic status of unknown egg samples collected from the different used-tire trading garages (Table 1, Additional file 2: Table S2).

## Results

### *Aedes* infestation along Panama’s primary transport network

Overall, we found *Aedes* mosquitoes present in 79 of 84 garages that trade used tires (90.01%) along the primary roads of Panama (Fig. 1, Additional file 3: Figure S1). Of those evaluated, only five garages lacked mosquitoes (Additional file 1: Table S1, Additional file 3: Figure S1). Since most negative garages were monitored when hurricane Otto was impacting the western provinces of Chiriquí, Bocas del Toro and Veraguas, bad weather conditions could have affected the normal oviposition cycle of the mosquitoes there. Furthermore, infestation rates were comparable for both species, with *Ae. aegypti* occurring alone or with *Ae. albopictus* in 45 (53.5%) garages, while *Ae. albopictus* was found, alone or with *Ae. aegypti*, in 50 (59.5%), suggesting that both mosquitoes readily oviposit in garages that trade used tires when available. However, they co-occurred in just 16 (19.1%) garages, depicting a disparate geographical distribution along the roads of Panama (Fig. 1, Table 1, Additional file 3: Figure S1). The spatial model of occurrence further supported that in garages that trade used tires along the highway, *Ae. aegypti* and *Ae. albopictus* co-occur less than expected by chance (Plt = 0.00002; Pgt = 1). *Aedes albopictus* occurred more often in rural western Panama, between the border of Costa Rica and Aguadulce in the province of Coclé, whereas *Ae. aegypti* was more frequently found along the highly-populated highways of central Panama, between Aguadulce and Panama City (Fig. 1, Table 1, Additional file 3: Figure S1). Although both species were found in the urban cities of Panama and Colón, we found used tires more frequently infested with *Ae. aegypti*. Furthermore, *Ae. albopictus* was the only mosquito found in rural areas along the trans-isthmian highway, which connects Panama and Colón (Fig. 1, Table 1, Additional file 3: Figure S1).

The presence of a garage was significantly linked to the density of roads across Panama (*R*^2^ = 0.4, *df* = 1, *P* = 0.001), whereby the presence of a garage was associated with an increase in the road density by a factor of 0.17. GLM analysis further revealed that the presence of *Ae. aegypti* is associated with an increase in road density by a log-odds of 0.44 (Wald Chi-Square = 9.57, *df* = 1, *P* = 0.002), while the presence of *Ae. albopictus* is associated with a decrease in road density by a log-odds of 0.36 (Wald Chi-square = 6.95, *df* = 1, *P* = 0.008) (Additional file 4: Table S3). The co-occurrence of *Aedes* species was not significantly linked to road density (Wald Chi-square = 0.01, *df* = 1, *P* = 0.933).

### MALDI-TOF-MS for mosquito egg species identification

**Fig. 2:**
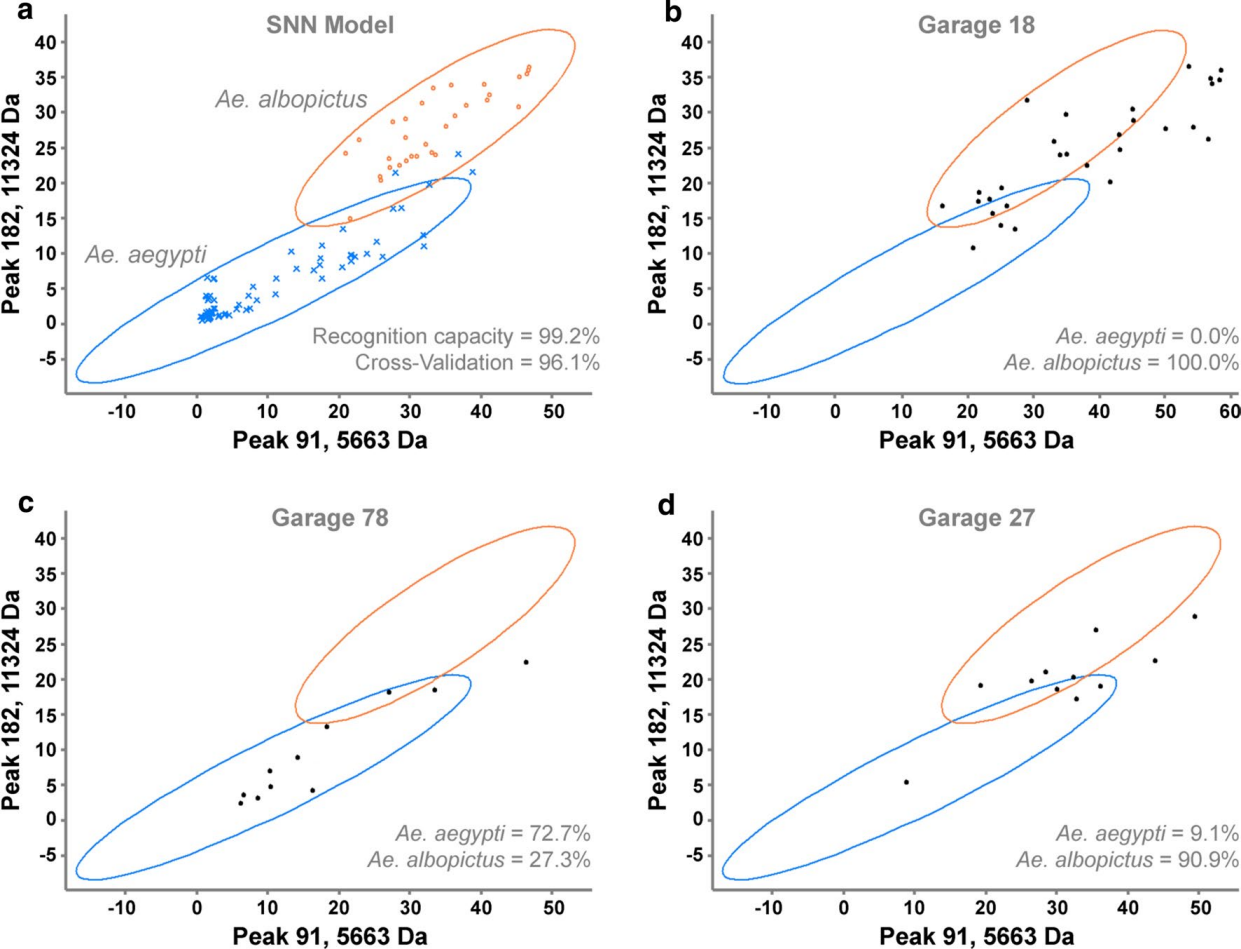
2D peak distribution graphs for the SNN model, showing the distribution of the two first (best separating) peaks using difference average peak statistics. **a** Ellipses represent the 95% confidence interval of the difference between the maximal and the minimal average peak areas/intensities for each reference mosquito egg class, *Ae. aegypti* (blue ellipse, X) and *Ae. albopictus* (orange ellipse, •). Classified spectra (black circles) are protein spectra identified to species level by the SNN algorithm, which are presented for three representative garages: garage 18 (**b**), which only had spectra corresponding to *Ae. albopictus* agreed with the conclusion drawn from emerged adult mosquitoes; garage 78 (**c**), which had co-existence of both species with a higher percentage of *Ae. aegypti*, agreed with corresponding emerged adult mosquitoes; and garage 27 (**d**), which had co-existence of both species due to a single spectrum identified as *Ae. aegypti*, contrary to the corresponding emerged adult mosquitoes, which concluded the presence of *Ae. albopictus* only. The rest of the classification graphs can be found in Additional file 2: Table S2

In total, 825 protein spectra were obtained from 312 eggs representing 17 garages distributed along Panama’s transport network (Table 1, Additional file 2: Table S2). Most samples identified using the MALDI-TOF-MS provided at least one suitable protein spectrum for analysis with a supervised neural network (SNN) classification algorithm. To identify the unknown specimens, we trained the SNN algorithm with *Aedes* reference mosquito egg spectra, which exhibited several mass peak differences between *Ae. aegypti* and *Ae. albopictus* (Additional file 5: Figure S2). The model had 99.2% recognition capacity, representing its ability to correctly classify the training data to a high proportion, and 96.1% species cross validation (Fig. 2a) representing its ability to classify a subset of known spectra not included in the training dataset.

In addition, the model established either the single existence of *Ae. albopictus* (Fig. 2b) or the coexistence of both *Ae. aegypti* and *Ae. albopictus* (Fig. 2c, d) in a particular garage, with close to 90% (15 of 17 garages) recognition power when compared to the reared adult mosquitoes from the respective egg counterpart (Table 1, Additional file 2: Table S2). In general, the spatial pattern of *Aedes* occurrence obtained with the MALDI-TOF-MS procedure mimics the one found using active surveillance and oviposition traps (Table 1). The presence of both *Ae. aegypti* and *Ae. albopictus* was confirmed in 13 garages using the SNN algorithm, while the single occurrence of *Ae. albopictus* was also established in 4 of 6 garages (Table 1, Additional file 2: Table S2). Two garages concluded the coexistence of both species with the MALDI-TOF-MS while rearing and taxonomic identification concluded the presence of *Ae. albopictus* only; however, garage 20 had a poor hatch rate with just a single egg successfully reared for taxonomic identification, demonstrating the difficulties encountered with the standard rearing procedure. Furthermore, MALDI-TOF-MS identified 10 of 11 spectra from garage 27 as *Ae. albopictus* (Fig. 2d) and just a single egg as *Ae. aegypti*, demonstrating a potential increase in resolution of this technique. In general, garages depicting coexistence were located in urban settings, while those with only *Ae. albopictus* were located in rural Panama (Table 1, Additional file 3: Figure S1).

## Discussion

The high rate of *Aedes* infestation uncovered in this study confirms the importance of the local transport network and the used-tire industry in Panama as a common habitat for the development of *Ae. aegypti* and *Ae. albopictus*, with trade providing ample opportunities for the dispersal of both mosquitoes. This results in important ramifications for the introduction, reintroduction and spread of new pathogens, new mosquito and viral strains, and new mutations arising from eradication methods. For instance, *Ae. albopictus* has become widespread across Panama since its introduction 13 years ago [9]. Such expansion cannot be easily explained by active flight alone, which is limited to ~1 km [30], whereas a passive human-assisted model incorporating the local transport system predicted its rapid expansion [9]. In support of this model, our study documents the novel presence of *Ae. albopictus* in several predicted regions of expansion, including the Azuero Peninsula, central Panama between Santiago and Panama City, and Darién province (Fig. 1, Additional file 3: Figure S1). These outcomes confirm the ability of *Ae. albopictus* to invade new geographical areas *via* human-aided dispersal along the local primary transport network and *via* the used-tire industry. This mode of dispersion would also explain the distribution of genetic diversity in this species across the Isthmus of Panama, characterized by widely distributed haplotypes resulting from multiple introductions [31].

The current distribution of *Aedes* across Panama can be explained by the co-action of multiple invasion events into the Isthmus of Panama, human-assisted dispersal through the primary road system and biological competition across different environmental conditions [9, 31]. Since *Ae. aegypti* and *Ae. albopictus* were found in spatial isolation more frequently than expected by chance, this suggests that either intra-specific competition for resources or differences in environmental preferences could influence their distribution in garages that trade used tires along the roads of Panama. Previously, it has been suggested that inter-specific resource competition in used-tire habitats leads to the displacement of *Ae. aegypti* by its dominant competitor, *Ae. albopictus*, in areas where it has recently invaded [32, 33]. In support of this, in the last two years we found *Ae. albopictus* as the sole species in locations of Panama where *Ae. aegypti* had been collected by the health authorities in previous years, which could suggest a pattern of species displacement. However, we cannot confirm this without thorough sampling across the entire region, including habitats other than used tires.

In previous studies, *Ae. albopictus* was more frequent in tires and container habitats in rural areas, while *Ae. aegypti* was dominant in warmer/urban environments [8, 32, 33]. We found that *Ae. albopictus* was more prevalent in rural Panama and associated with lower road density while *Ae. aegypti* was more frequent along highly-populated highways, more common in garages surrounded by higher road density and dominant in Panama City. That these species are able to coexist in the most urbanised regions of Panama, as in other areas of the world, suggests that differences in species distributions do not result from a simple species replacement by the dominant competitor, but from a combination of factors. The environmental conditions which impact competition between *Aedes* species are yet to be identified, but life history traits (i.e. development time and egg survivorship), availability of hosts and the distribution of favorable habitats could be the focus of future investigation [8, 34]. Whether the two species actively avoid laying eggs in the same tires or whether one outcompetes the other *in situ* is yet to be addressed within Panama. However, if *Ae. albopictus* is able to spread and replace *Ae. aegypti* as the prevalent vector throughout Panama’s interior, this expansion could be facilitated by dispersal opportunities offered by the used-tire industry. Our finding that *Ae. aegypti* and *Ae. albopictus* are not evenly distributed might support the need for various control strategies to decrease arboviral transmission according to the presence of different vectors in ecologically distinct areas of Panama.

Utilising the transport system could provide an interesting means through which to control both mosquitoes and disease transmission. For example, infection with *Wolbachia* bacteria has been proposed to control mosquito populations, firstly because infection can reduce the transmission of arboviruses and secondly, through exploiting the mechanism of cytoplasmic incompatibility to produce unviable offspring and/or drive *Wolbachia* infection through mosquito populations [35]. Utilizing known migration routes allows circumvention of the ecological and geographical barriers that can hamper this gene drive system [36]. Recently, both *Ae. aegypti* and *Ae. albopictus* were found naturally infected with *Wolbachia* in Panama, including *Ae. albopictus* with the strain wAlb B from containers and used tires widespread across Panama, and one individual of *Ae. aegypti* originating from a container habitat in provincial Panama. The potential for this strain to control both *Aedes* species in Panama, and its impact on viral transmission has not yet been fully assessed [37], but provides an interesting opportunity given its natural occurrence in mosquitoes developing in used tires. A future focus on determining the rates of gene flow along the highway, including the distribution of knockdown insecticide resistance haplotypes and natural *Wolbachia* infection, would further clarify the role of human-assisted dispersal in the introduction of novel genomic and pathogenic material into mosquito populations across Panama.

Overall, our results support the idea that garages that trade used tires on the highway should be targeted for surveillance and vector control in Panama, and that passive migration by human-assisted transport should be factored into models predicting disease outbreaks. The MALDI-TOF-MS procedure we applied to identify *Aedes* eggs can serve this purpose, facilitating the rapid assessment of mosquito infestation and species coexistence along the world’s trade networks. In addition, this approach could aid ecological studies and routine surveillance carried out by the health authorities within residential areas, including the evaluation of insecticide or other population control treatments and the monitoring of invasive introduction at areas of commerce such as ports. The MALDI-TOF approach reached the same conclusion as the traditional approach in 90% of cases, yet it is more time efficient and bypasses inaccuracy introduced by the differential hatching success of mosquitoes reared under laboratory conditions. Future improvements to the mathematical algorithm through the incorporation of dynamic learning to the SNN [38] or support vector machine (SVM) learning [39] could increase species recognition power. The MALDI-TOF approach to species identification of mosquito eggs has advantages over molecular methods based on polymerase chain reaction [40–42], as it is more cost effective (< US$0.5 per sample), can be completed to species identification within a few hours and is not impacted by the quality of DNA, genetic variation among geographical populations and/or species introgression.

## Conclusions

That both *Ae. aegypti* and *Ae. albopictus* mosquitoes utilise used tires imported from around the world [32] and are transported along Panama’s primary transport network [9] raises concerns given the propensity for these *Aedes* species to invade and establish in geographically naïve areas, where they may exchange alleles conferring insecticide resistance and/or influencing vector competence [43, 44]. Insecticide sprays are widely applied across Panama but are unlikely to prove an effective long-term population control strategy when re-invasion of *Ae. aegypti* and *Ae. albopictus* from other areas is facilitated by passive transport. To guarantee the sustainability of mosquito control in Panama, the current surveillance system must consider routes of potential re-infestation and employ innovative ways to identify mosquitoes quickly and effectively. The accurate identification of *Ae. aegypti* and *Ae. albopictus* through mass spectrometry analysis with egg samples will be key to this end, allowing for the rapid assessment of species infestation and coexistence, while affording time and cost advantages over traditional vector surveillance methods. Long-term monitoring using this scalable method could allow for the estimation of mosquito population densities, an important parameter for disease prediction models.

## Additional files

**Additional file 1: Table S1.** Complete list of used-tire trading garages mapped along the highways of Panama with information on the road density at the recorded geographical location, the species present and the number of individuals recorded through active surveillance (AS) and oviposition traps (OVT). Samples from garages marked with (*) were subjected to analysis with MALDI-TOF-MS.

**Additional file 2: Table S2.** Complete spreadsheet of used-tire trading garages selected for mosquito egg mass spectra generation with their respective SNN model prediction.

**Additional file 3: Figure S1.** The presence and absence of *Aedes* mosquitoes recorded along the major transport highways of Panama. Image created with ArcMap version 10.6 using original data and shapefiles obtained from the GIS Laboratory, Smithsonian Tropical Research Institute 2011 (https://strimaps.si.edu/portal/home/).

**Additional file 4: Table S3.** Results of the Generalised Linear Model. Analysis was performed using a Poisson distribution with a logit link to test whether the presence of only *Ae. aegypti*, only *Ae. albopictus* or the co-occurrence of both species was linked to the density of roads. The density of roads was the dependent variable in analyses.

**Additional file 5: Figure S2.** Representative mass spectra of reference *Ae. aegypti* and *Ae. albopictus* mosquito eggs used to train the Supervised Neural Network (SNN) classification algorithm. Spectra were collected with a MALDI-TOF-MS in the range of 2,000 to 20,000 m/z in positive ion mode, using α-Cyano-4-hydroxycinnamic acid (HCCA) matrix. * highlight representative peaks with the highest difference average selected for species classification. The profile peaks selected for species classification were based on all reference spectra from both species and are given in Additional file 2: Table S2.

## Data Availability

All data supporting the conclusions of this article are provided within the article and its additional files.

## Abbreviations

MALDI-TOF-MS: matrix-assisted laser desorption/ionization time-of-flight mass spectrometry
SNN: supervised neural network
HCCA: α-Cyano-4-hydroxycinnamic acid matrix
AS: active surveillance
OVT: oviposition traps
