# Supplementary figures and images for: High infestation of invasive Aedes mosquitoes in used tires along the local transport network of Panama
Source: Parasit Vectors. 2019 May 27;12:264. doi: 10.1186/s13071-019-3522-8 (PMC6537307; doi:10.1186/s13071-019-3522-8)

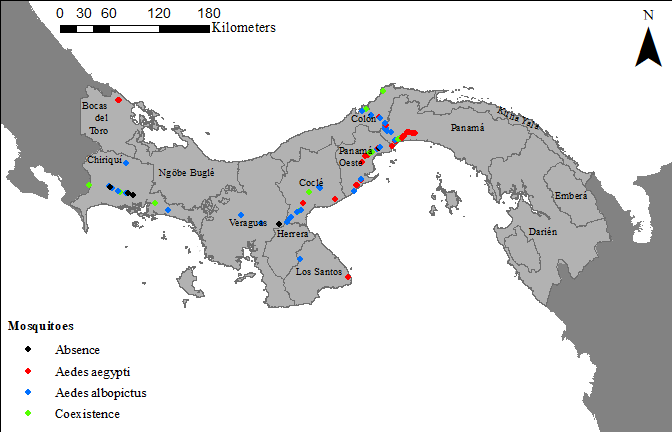

Supplement: Supplementary file 3 — Additional file 3: Figure S1. The presence and absence of Aedes mosquitoes recorded along the major transport highways of Panama. Image created with ArcMap version 10.6 using original data and shapefiles obtained from the GIS Laboratory, Smithsonian Tropical Research Institute 2011 (https://strimaps.si.edu/portal/home/). [file 13071_2019_3522_MOESM3_ESM.png]

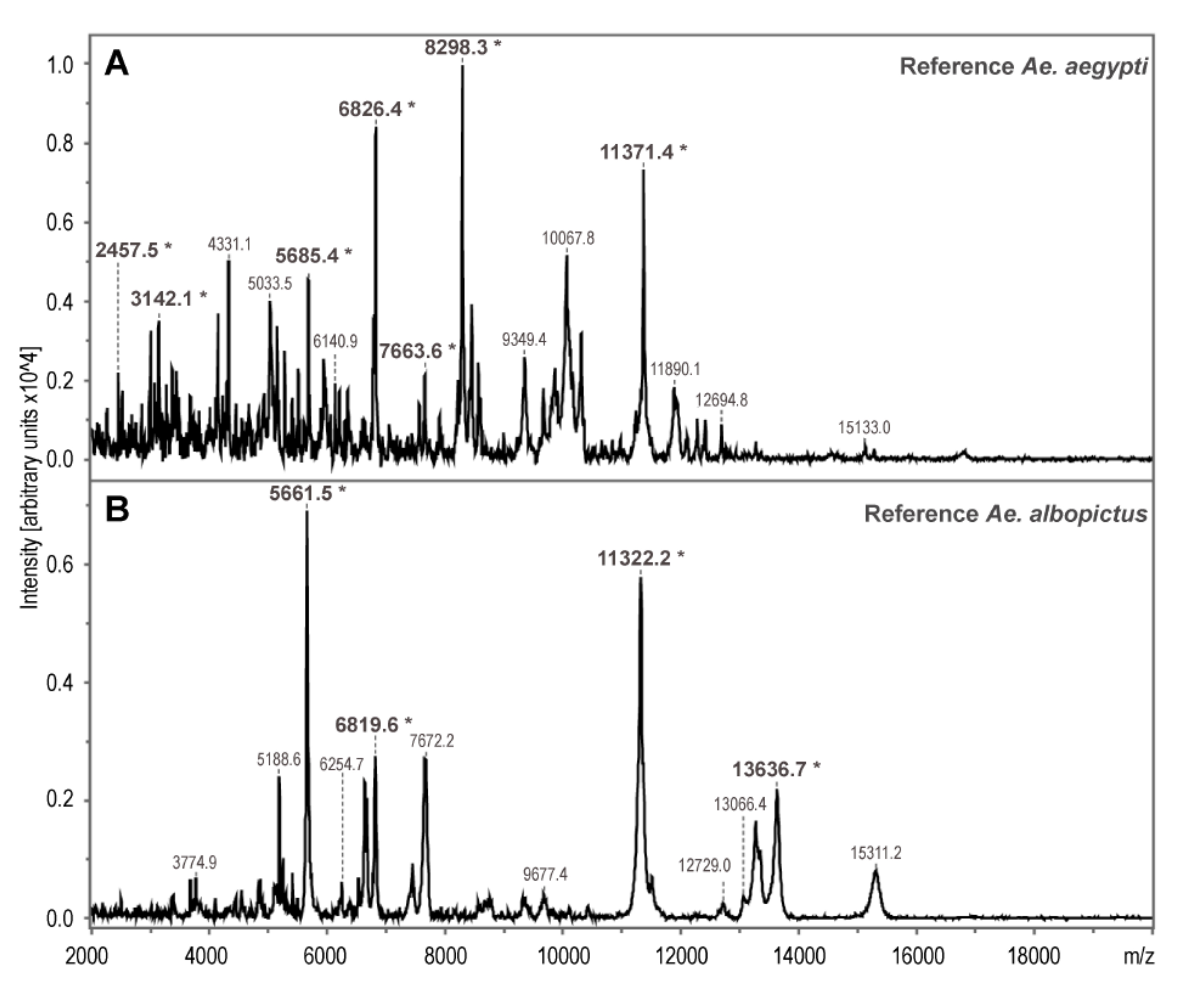

Supplement: Supplementary file 5 — Additional file 5: Figure S2. Representative mass spectra of reference Ae. aegypti and Ae. albopictus mosquito eggs used to train the Supervised Neural Network (SNN) classification algorithm. Spectra were collected with a MALDI-TOF-MS in the range of 2,000 to 20,000 m/z in positive ion mode, using α-Cyano-4-hydroxycinnamic acid (HCCA) matrix. * highlight representative peaks with the highest difference average selected for species classification. The profile peaks selected for species classification were based on all reference spectra from both species and are given in Additional file 2: Table S2. [file 13071_2019_3522_MOESM5_ESM.jpg]
